# AF4 and AF4-MLL mediate transcriptional elongation of 5-lipoxygenase mRNA by 1, 25-dihydroxyvitamin D_3_

**DOI:** 10.18632/oncotarget.4703

**Published:** 2015-07-23

**Authors:** Khalil Ahmad, Bastian Scholz, Ricardo Capelo, Ilona Schweighöfer, Astrid Stefanie Kahnt, Rolf Marschalek, Dieter Steinhilber

**Affiliations:** ^1^ Institute of Pharmaceutical Chemistry / ZAFES Goethe University Frankfurt; ^2^ Institute of Pharmaceutical Biology / ZAFES, Goethe University Frankfurt, Germany

**Keywords:** 5-lipoxygenase, MLL, AF4, calcitriol, HDAC

## Abstract

The human 5-lipoxygenase (5-LO), encoded by the *ALOX5* gene, is the key enzyme in the formation of pro-inflammatory leukotrienes. *ALOX5* gene transcription is strongly stimulated by calcitriol (1α, 25-dihydroxyvitamin D_3_) and TGFβ (transforming growth factor-β). Here, we investigated the influence of MLL (activator of transcript initiation), AF4 (activator of transcriptional elongation) as well as of the leukemogenic fusion proteins MLL-AF4 (ectopic activator of transcript initiation) and AF4-MLL (ectopic activator of transcriptional elongation) on calcitriol/TGFβ-dependent 5-LO transcript elongation. We present evidence that the AF4 complex directly interacts with the vitamin D receptor (VDR) and promotes calcitriol-dependent *ALOX5* transcript elongation. Activation of transcript elongation was strongly enhanced by the AF4-MLL fusion protein but was sensitive to Flavopiridol. By contrast, MLL-AF4 displayed no effect on transcriptional elongation. Furthermore, HDAC class I inhibitors inhibited the ectopic effects caused by AF4-MLL on transcriptional elongation, suggesting that HDAC class I inhibitors are potential therapeutics for the treatment of t(4;11)(q21;q23) leukemia.

## INTRODUCTION

Human 5-lipoxygenase (5-LO), which is encoded by the *ALOX5* gene, catalyzes the first two steps in the biosynthesis of the leukotrienes from arachidonic acid. Leukotrienes are a part of the innate immune system but are also associated with inflammatory, allergic and cardiovascular diseases as well as certain types of cancer [1].

The human *ALOX5* gene consists of 14 exons and 13 introns, named as introns A-M, respectively [2]. The *ALOX5* promoter contains eight GC-boxes but lacks TATA and CAAT boxes, and thus, resembles promoters of housekeeping genes although 5-LO is mainly expressed in leukocytes [1, 3]. 5-LO mRNA expression is regulated at the level of transcript initiation and elongation. The *ALOX5* promoter can be activated by the pan-histone deacetylase (HDAC) inhibitor Trichostatin A (TSA) and by class I HDAC inhibitors (HDACi) [4, 5], an effect which depends mainly on the recruitment of the transcription factor Sp1 to a single cognate binding site close to the transcriptional start site [6]. Induction of 5-LO mRNA transcription by TSA also correlates with MLL activation and the subsequent upregulation of H3K4me3 signatures at the *ALOX5* promoter [5]. MLL-dependent *ALOX5* promoter activation is stimulated by VDR/RXR as well as SMADs in a ligand-independent manner.

By contrast, 5-LO mRNA expression has been described to be strongly stimulated by the respective ligands, calcitriol and TGFβ [7]. These ligand-dependent effects are, however, not mediated via the *ALOX5* promoter but are due to enhanced transcriptional elongation [8]. Transcriptional elongation is induced by subsequent phosphorylation steps at Ser-5 by TFIIH and finally at Ser-2 and Thr-4 residues of the repetitive C-terminal domain (CTD: 52 repeats) of RNA polymerase II (RNAPII) by the positive transcription elongation factor b (P-TEFb) [9–11], a dimeric protein consisting of CDK9 and Cyclin T1. P-TEFb is recruited to active promoters as part of the super elongation complexes that are composed by either AF4 (AFF1) or AF5 (AFF4), and include - among other proteins - AF9, AF10, ELL and the two histone methyltransferases NSD1 and DOT1L [12–15]. The AF4 protein recruits P-TEFb from 7SK RNP inhibitory complexes and stimulates transcriptional elongation by increasing P-TEFb-mediated Ser-2 phosphorylation of RNAPII [12]. Of interest, the *AF4* gene is frequently involved in t(4;11)(q21;q23) reciprocal chromosomal translocations with the *MLL* gene [16]. The resulting fusion proteins AF4-MLL (der4) and MLL-AF4 (der11) lead to development and maintenance of high-risk acute lymphoblastic leukemia (ALL) [14, 17–19].

Previously, we could demonstrate that MLL-AF4 is a constitutive activator of gene transcription and induces transcript initiation [5]. In this study, we investigated the influence of both wild-type AF4 and MLL, as well as of the t(4;11) fusion proteins AF4-MLL and MLL-AF4 on transcriptional elongation using the recently discovered calcitriol/TGFβ-dependent elongation of *ALOX5* transcripts as an experimental model system. We found that the AF4 complex directly interacts with the VDR (vitamin D receptor) and acts on 5-LO transcript elongation. Not surprisingly, the AF4-MLL fusion protein mimics the function of the AF4 complex, however, in a much more enhanced and stringent way. Class I HDACi inhibited AF4-MLL-induced 5-LO transcriptional elongation, which indicates that these inhibitors are able to attenuate the aberrant epigenetic activity of AF4-MLL. Thus, HDACi are not only blocking the actions deriving from MLL-AF4 [5], but also that of AF4-MLL. Therefore, our results suggest that class I HDAC inhibition might be an interesting option for the therapy of t(4;11)(q21;q23) leukemias.

## RESULTS

### MLL-AF4 activates the 5-LO promoter whereas AF4-MLL leads to calcitriol/TGFβ-dependent 5-LO transcript elongation

**Figure 1:**
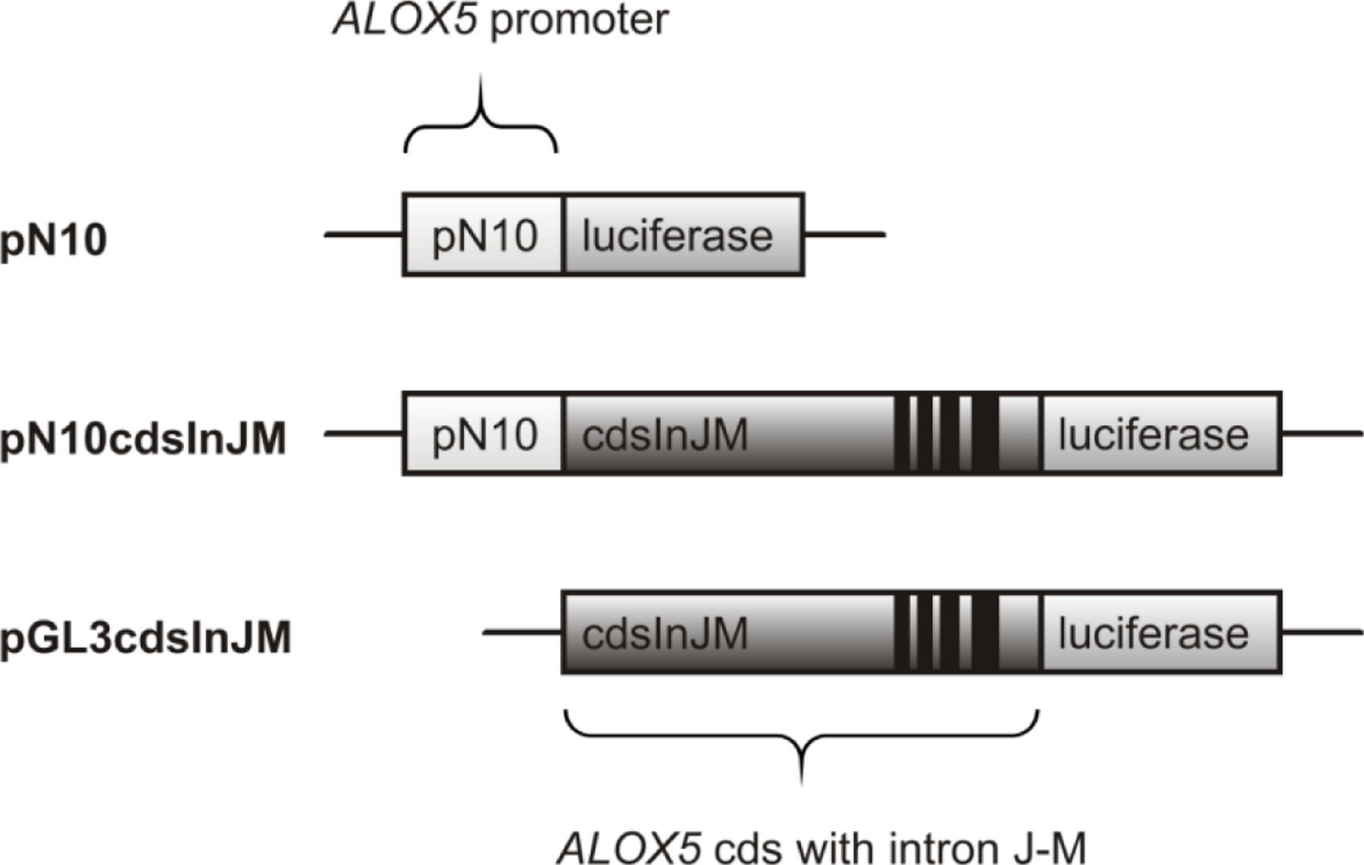
Schematic representation of applied reporter gene constructs in the reporter gene assay experiments

**Figure 2:**
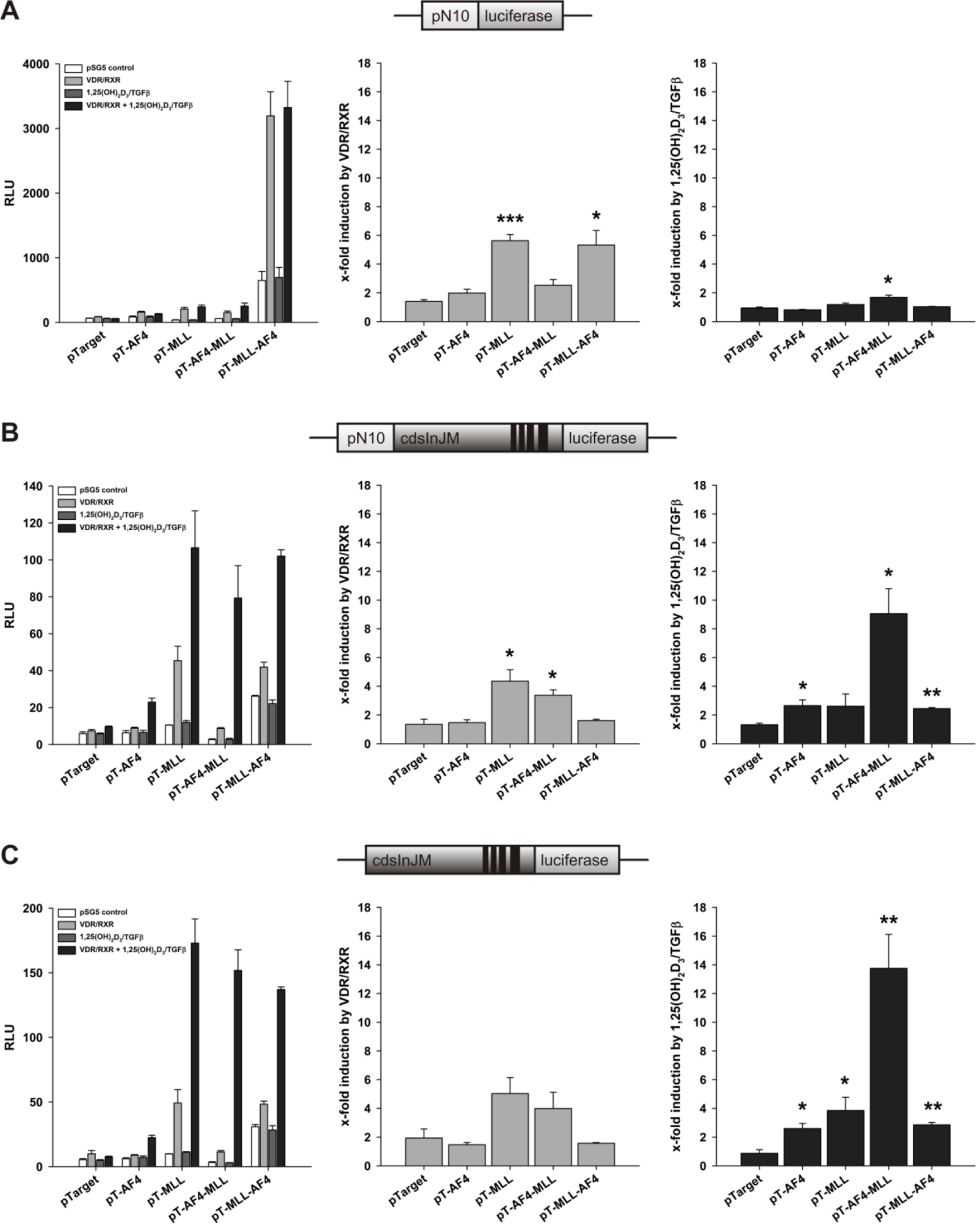
Effect of AF4, MLL, AF4-MLL and MLL-AF4 overexpression on VDR/RXR- and calcitriol/TGFβ-dependent response of the reporter gene constructs A. pN10 B. pN10cdsInJM and C. pGL3cdsInJM In order to study inducibility of the constructs by calcitriol/TGFβ, HeLa cells were co-transfected either with the pSG5 control vector or with the expression vectors for the nuclear receptors VDR and RXR. 16 hours after transfection, cells were incubated without or with calcitriol (50 nM) and TGFβ (1 ng/ml). After 40 hours, luciferase activity was determined. Results are given as RLU (left panels) which include normalization to transfection efficiency and as x-fold induction by VDR/RXR over pSG5 control (middle panels) or x-fold induction by calcitriol/TGFβ over VDR/RXR (right panels). Each experiment was performed in triplicate. Results are presented as mean + S.E.M. of three independent experiments. Two-tailed *t*-test was performed to determine significant differences in relation to co-transfection of the backbone vector pTarget, indicated by asterisks (*P < 0.05, **P < 0.01, ***P < 0.001).

In order to study the effects of MLL and its oncogenic counterparts on ALOX5 transcript initiation and elongation, HeLa cells were transiently transfected with the pN10 (Figures 1, 2A), pN10cdsInJM (Figures 1, 2B) or the pGL3cdsInJM (Figures 1, 2C) luciferase reporter gene construct and co-transfected either with pTarget (empty vector control) or pTarget-based expression vectors for AF4, MLL, AF4-MLL or MLL-AF4. The 5-LO promoter/reporter gene vector pN10 contains the *ALOX5* promoter sequence from −778 to +53 (relative to the transcriptional start site) fused to the luciferase reporter gene. Activity of this construct is not elongation controlled because it was not sensitive to Flavopiridol treatment (see also Figure 8B). Therefore, this construct allows to measure transcript initiation selectively. The second reporter gene construct pN10cdsInJM and the third promoterless construct pGL3cdsInJM contain both the coding sequence as well as the last four introns of the *ALOX5* gene, a region which has already been shown to be strictly elongation controlled [8].

As already shown previously, in the absence of co-transfected VDR/RXR, *ALOX5* promoter activity was strongly induced by MLL-AF4, whereas the activity was unaffected by AF4, MLL and AF4-MLL expression (Figure 2A, left panel). Interestingly, VDR/RXR coexpression strongly induced *ALOX5* promoter activity together with MLL and MLL-AF4 but less with AF4 and AF4-MLL (Figure 2A, middle panel). These effects are not dependent on calcitriol/TGFβ (Figure 2A, right panel). Taken together, the results confirm our previous observation, that MLL-AF4 strongly stimulates 5-LO promoter activity. Additionally, we found here that VDR/RXR support 5-LO promoter activation by MLL and MLL-AF4 in a ligand-independent manner.

Next, we analyzed the reporter gene constructs pN10cdsInJM (Figure 2B) and pGL3cdsInJM (Figure 2C). Similar to the pN10 reporter construct, MLL-AF4 expression induced activity of both reporter gene constructs (Figure 2B and 2C, left panels). However, the absolute activity was much lower than with pN10. Interestingly, VDR/RXR coexpression enhanced the effects of MLL and AF4-MLL, but not MLL-AF4 in both elongation controlled reporter gene constructs (Figure 2B and 2C, middle panels) indicating that this effect can be attributed to either the N-terminal portion of AF4 or the C-terminal portion of MLL.

Of note, in contrast to pN10, we observed strong ligand dependent effects with both elongation-controlled constructs (Figure 2B and 2C, right panels). Thus, calcitriol/TGFβ stimulated reporter gene activity up to 4-fold when AF4, MLL or MLL-AF4 were co-expressed. The most remarkable effect was obtained in the presence of AF4-MLL, where a strong, up to 14-fold VDR/RXR-dependent induction of reporter gene activity was observed by calcitriol/TGFβ (Figure 2B and 2C, right panels). The data suggest that transcript elongation, but not initiation is stimulated by calcitriol/TGFβ and that AF4-MLL strongly enhances the calcitriol/TGFβ effects.

### SMAD effects

**Figure 3:**
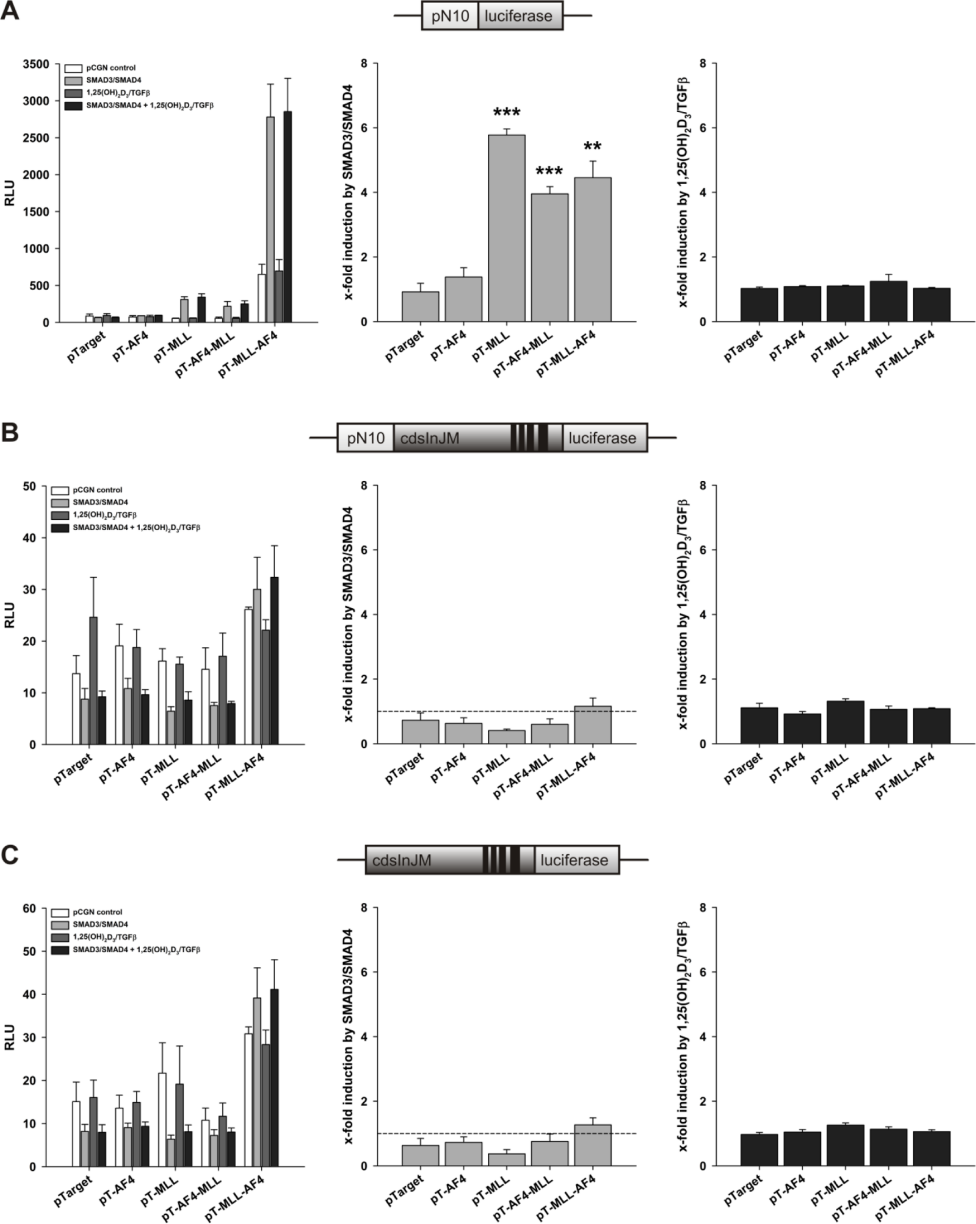
Effect of AF4, MLL, AF4-MLL and MLL-AF4 overexpression on SMAD3/SMAD4- and calcitriol/TGFβ-dependent response of the reporter gene constructs A. pN10 B. pN10cdsInJM and C. pGL3cdsInJM In order to study inducibility of the constructs by calcitriol/TGFβ, HeLa cells were co-transfected either with the pCGN control vector or with the expression vectors for the nuclear receptors SMAD3 and SMAD4. 16 hours after transfection, cells were incubated without or with calcitriol (50 nM) and TGFβ (1 ng/ml). After 40 hours, luciferase activity was determined. Results are given as RLU (left panels) which include normalization to transfection efficiency and as x-fold induction by SMAD3/SMAD4 over pCGN control (middle panels) or x-fold induction by calcitriol/TGFβ over SMAD3/SMAD4 (right panels). Each experiment was performed in triplicate. Results are presented as mean − S.E.M. of three independent experiments. Two-tailed *t*-test was performed to determine significant differences in relation to co-transfection of the backbone vector pTarget, indicated by asterisks (*P < 0.05, **P < 0.01, ***P < 0.001).

Since calcitriol as well as TGFβ induce cellular 5-LO mRNA expression, we studied the role of SMAD3/SMAD4 in all three reporter systems (compare Figure 2). In contrast to VDR/RXR, expression of SMAD3 and SMAD4 only supported the stimulation of pN10 (pure promoter construct) by MLL, AF4-MLL and MLL-AF4 (Figure 3A), but not from the elongation controlled plasmids pN10cdsInJM and pGL3cdsInJM (Figure 3B and 3C). Moreover, we did not see any ligand-dependent effects under our assay conditions (Figure 3A–3C). The data suggest that SMADs enhance transcript initiation but do not stimulate transcript elongation.

### Calcitriol is the critical ligand for VDR/RXR mediated induction by AF4-MLL

**Figure 4:**
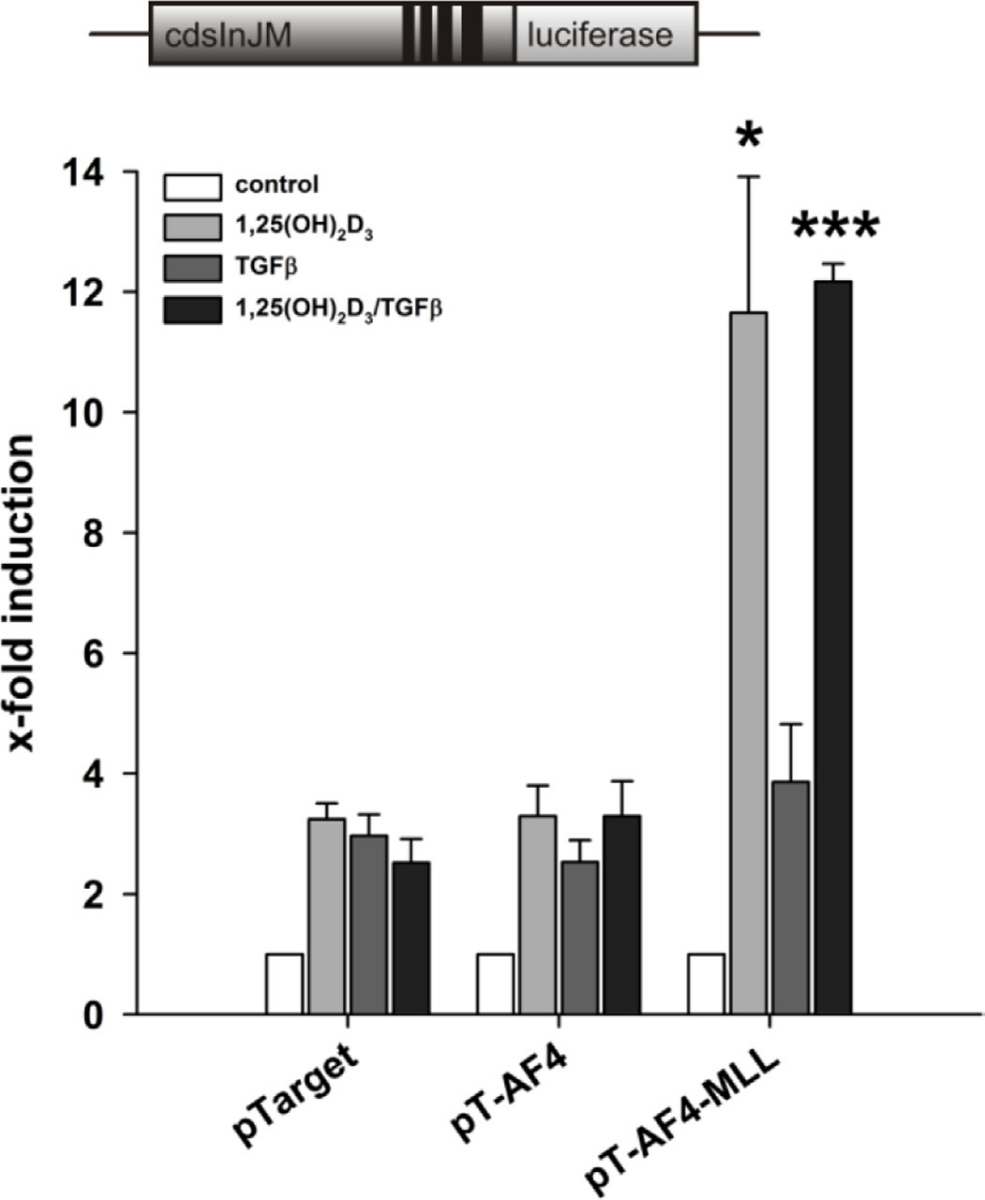
Contribution of calcitriol and TGFβ to the AF4-MLL-dependent reporter gene activation by calcitriol/TGFβ HeLa cells were transfected with the pGL3cdsInJM reporter gene construct as well as the expression vectors for the nuclear receptors VDR and RXR and were co-transfected either with the pTarget control vector or the expression vectors for AF4 or AF4-MLL as indicated. 16 hours after transfection, cells were incubated without or with calcitriol (50 nM) and/or TGFβ (1 ng/ml). After 40 hours, luciferase activity was determined and results are given as x-fold induction over untreated cells. Each experiment was performed in triplicate. Results are presented as mean − S.E.M. of three independent experiments. Two-tailed *t*-test was performed to determine significant differences in relation to co-transfection of the backbone vector pTarget, indicated by asterisks (*P < 0.05, **P < 0.01, ***P < 0.001).

*ALOX5* is known for long as a calcitriol/TGFβ responsive gene [7, 20–22]. Since calcitriol/TGFβ strongly stimulate 5-LO transcript elongation, we investigated the effects of either TGFβ or calcitriol on the AF4-MLL-mediated *ALOX5* gene induction in order to identify whether these ligand-dependent effects are due to calcitriol or TGFβ or to the combination of both agents. HeLa cells were transiently transfected with pGL3cdsInJM, the VDR/RXR expression plasmids and either with empty vector (pTarget) or the expression vectors for AF4 or AF4-MLL. Subsequently, we studied the effects of calcitriol, TGFβ or the combination of both compounds. As shown in Figure 4, combined incubation with calcitriol/TGFβ led to a highly significant induction of reporter gene activity in the presence of AF4-MLL. Interestingly, calcitriol alone exhibited a similar induction, whereas TGFβ showed no effect under these conditions. The data suggest that stimulation of 5-LO transcript elongation is due to vitamin D- and not TGFβ-signalling.

### Inhibition of proteasomal degradation attenuates the difference between AF4 and AF4-MLL

**Figure 5:**
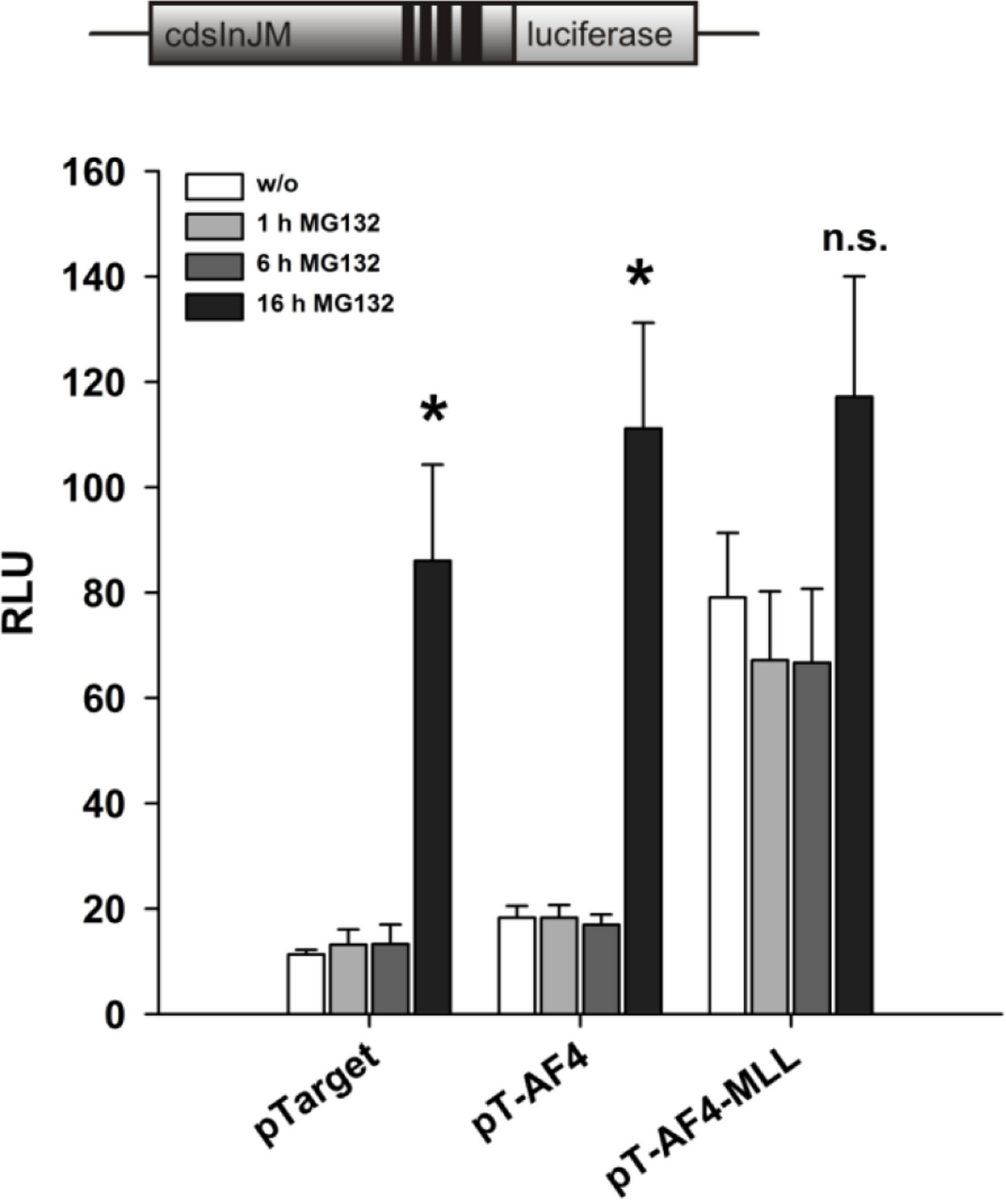
Effect of the proteasome inhibitor MG132 on reporter gene activation by AF4 and AF4-MLL HeLa cells were transfected with the pGL3cdsInJM reporter gene construct and the expression vectors for the nuclear receptors VDR and RXR and were co-transfected either with the pTarget control vector or the expression vectors for AF4 or AF4-MLL. 16 hours after transfection, cells were incubated with calcitriol (50 nM) and TGFβ (1 ng/ml). Cells were treated with MG132 (5 μM) for 1, 6 and 16 hours. 40 hours after transfection, luciferase activity was determined and the results are given as RLU which include normalization to transfection efficiency. Each experiment was performed in triplicate. Results are presented as mean − S.E.M. of three independent experiments. Two-tailed *t*-test was performed to determine the significance in relation to MG132 untreated cells, indicated by asterisks (*P < 0.05, **P < 0.01, ***P < 0.001).

Under physiological conditions, the AF4 protein is produced, forms the AF4 complex, activates the process of transcriptional elongation, and is subsequently rapidly degraded via binding to the E3 ligases SIAH1 or SIAH2, respectively. The proteasomal degradation pathway is so fast that endogenous AF4 cannot be visualized in Western blot experiments [23]. In order to check whether the lower induction of transcript elongation by AF4 as compared to AF4-MLL is due to proteasomal degradation of AF4 and a subsequent lower cellular expression level, we transiently transfected HeLa cells with pGL3cdsInJM, VDR/RXR expression plasmids and expression vectors for AF4 or AF4-MLL. The transfected cells were incubated with calcitriol/TGFβ and with the proteasome inhibitor MG132 (5 μM) for different time periods (1 h, 6 h and a maximum of 16 h). The treatment of HeLa cells with MG132 for 16 hours resulted in an increase in AF4-mediated activities leading to similar effects as with AF4-MLL (Figure 5). Furthermore, treatment with MG132 also stimulated the activity of empty vector transfected cells, due to an about 4-fold stabilization of endogenous AF4 protein (measured with the AFF1 ELISA Kit, data not shown). The data suggest that the N-terminal part of AF4 mediates stimulation of transcript elongation and that the lower activity of AF4 compared to the oncogenic fusion protein AF4-MLL is due to its rapid proteasomal degradation, while the AF4-MLL fusion protein is not being degraded via the proteasomal pathway [23].

### Knockdown of AF4 reduces basal activity of transcript elongation

**Figure 6:**
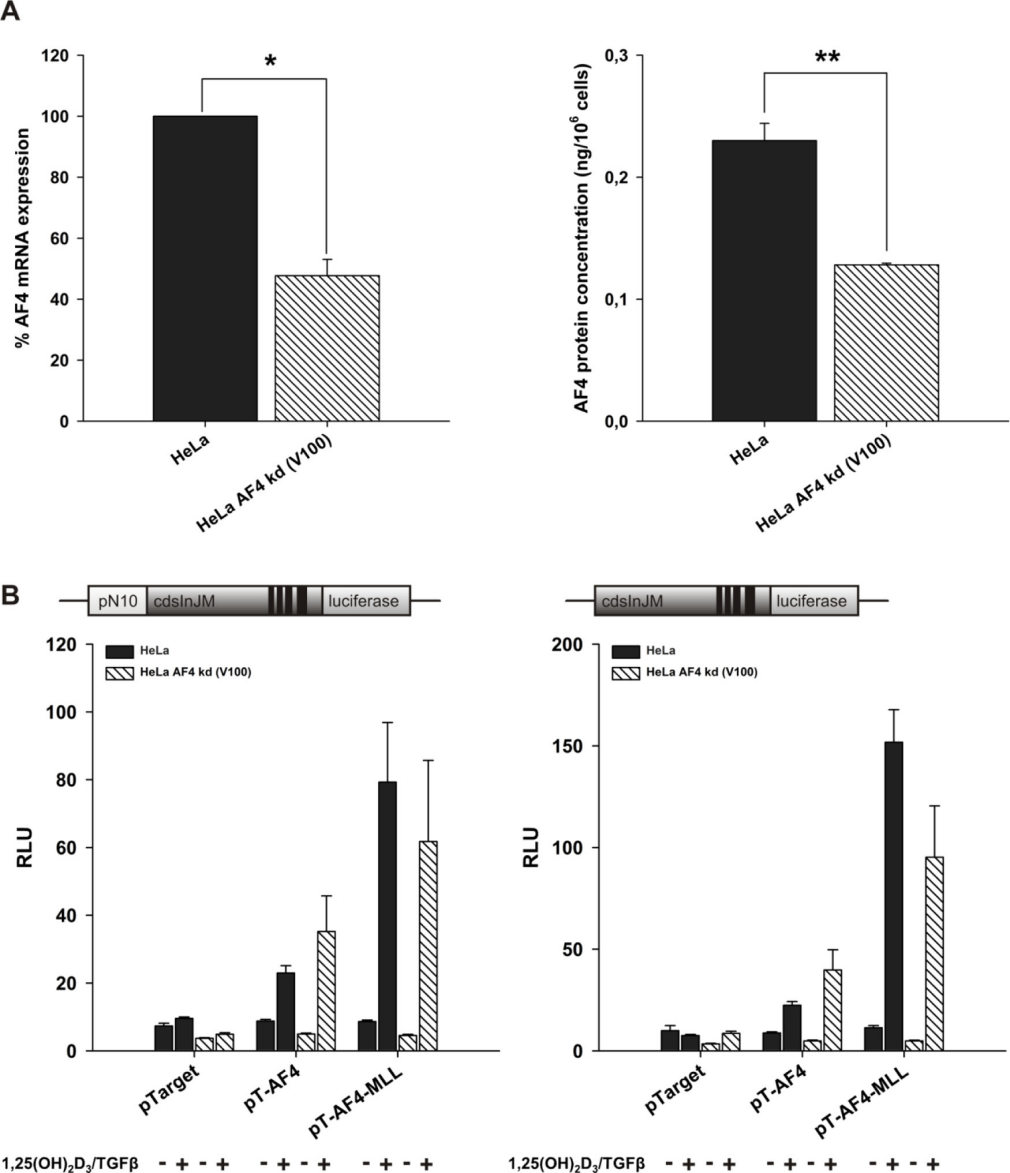
Effect of stable AF4 knockdown in HeLa cells **A.** AF4 knockdown cells were generated with lentiviral particles, produced by co-transfection of HEK293T cells with the shRNA plasmid for AF4, pCMV-dR8.91 and pMD2.G. Knockdown efficiency of AF4 was checked by qPCR, using β-actin as housekeeping gene, and viaAFF1 ELISA Kit in three independent experiments, respectively. One sample *t*-test was performed to determine the significance of AF4 knockdown on mRNA level (left graph) and two-tailed *t*-test on protein level (right graph) (*P < 0.05, **P < 0.01, ***P < 0.001). **B.** HeLa wild type and HeLa AF4 knockdown cells were co-transfected with the pN10cdsInJM or pGL3cdsInJM reporter gene construct, pTarget or the expression vectors for AF4 or AF4-MLL and with the expression vectors for the nuclear receptors VDR and RXR. 16 hours after transfection, cells were incubated without or with calcitriol (50 nM) and TGFβ (1 ng/ml) as indicated. After 40 hours, luciferase activity was determined and is given as RLU which include normalization to transfection efficiency. Each experiment was performed in triplicate. Results are presented as mean + S.E.M. of three independent experiments.

To address the role of AF4 in *ALOX5* transcript elongation, a stable knockdown of endogenous AF4 was performed. As shown in Figure 6A the shRNA-mediated knockdown of AF4 resulted in a reduction of about 50% at the mRNA level, and a reduction of about 45% at the protein level. AF4 knockdown cells led to reduced cell viability and proliferation rate when compared with wild-type HeLa cells (data not shown). Knockdown cells transfected with pGL3cdsInJM and VDR/RXR displayed a significantly reduced basal reporter activity compared to wild-type cells, thus supporting that AF4 is crucial for 5-LO transcript elongation. In contrast, ectopic overexpression of AF4 or AF4-MLL and a subsequent incubation with calcitriol/TGFβ revealed no differences between AF4 knockdown and wild-type HeLa cells (Figure 6B), indicating that transfected AF4 or AF4-MLL was able to complement the knockdown of endogenous AF4.

### AF4 interacts with the VDR

**Figure 7:**
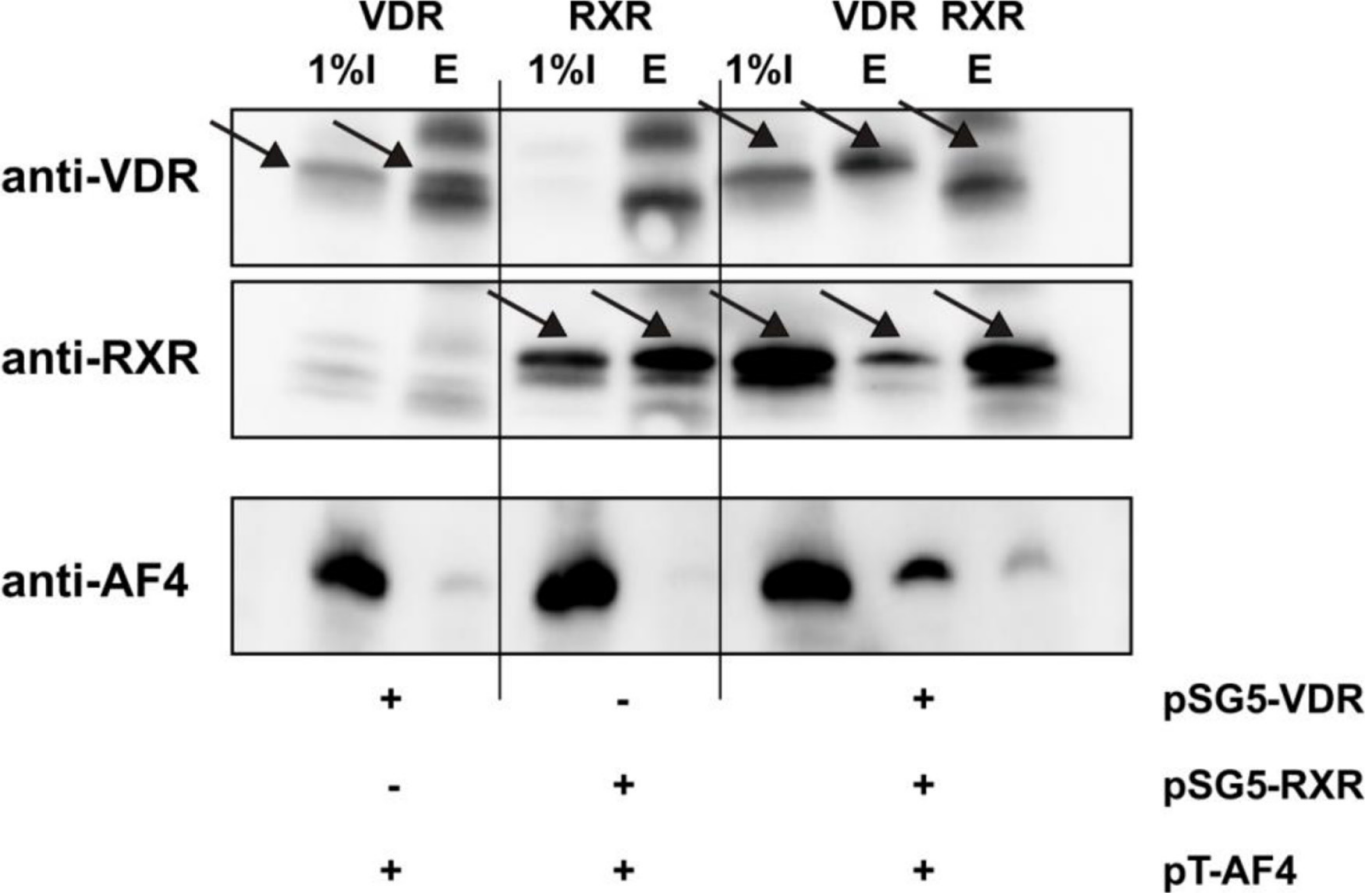
Co-immunoprecipitation of AF4 and VDR/RXR HEK293T cells were co-transfected with the expression vectors for AF4 and the nuclear receptors VDR and/or RXR as indicated. Then, cells were cultured for 48 hours. After harvest and cell lysis, immunoprecipitation was performed with VDR or RXR antibodies as indicated. Subsequently, supernatants (I) and bead eluates (E) were analyzed by Western blot analysis for VDR, RXR and AF4. Figure illustrates one representative Co-IP of three independent experiments.

Since we observed a VDR/RXR-dependent induction of transcription by calcitriol that was strongly stimulated by AF4 and AF4-MLL, we wondered whether VDR/RXR is able to directly interact with AF4. In order to verify that the VDR/RXR heterodimer and AF4 are interaction partners, we performed a Co-IP experiment. HEK293T cells were transfected with the expression plasmids for AF4 as well as the nuclear receptors VDR and/or RXR. Cells lysates were incubated with an antibody against VDR or RXR and immunoprecipitation was performed with Protein-G-Agarose. Subsequently performed Western blot experiments revealed that AF4 was not present in the precipitates of RXR-transfected cells, but was weakly present in precipitates of VDR-transfected cells. Interestingly, immunoprecipitates from VDR/RXR transfected cells display a prominent AF4 signal after immunoprecipitation with VDR antibody whereas a weaker signal was obtained when the RXR antibody was used, suggesting that VDR/RXR heterodimers serve as an AF4 or AF4 complex docking site and that VDR could be the component of the VDR/RXR heterodimer which mediates this interaction (Figure 7).

### Class I HDAC inhibitors and the CDK9 inhibitor Flavopiridol diminish AF4-MLL mediated transcript elongation

**Figure 8:**
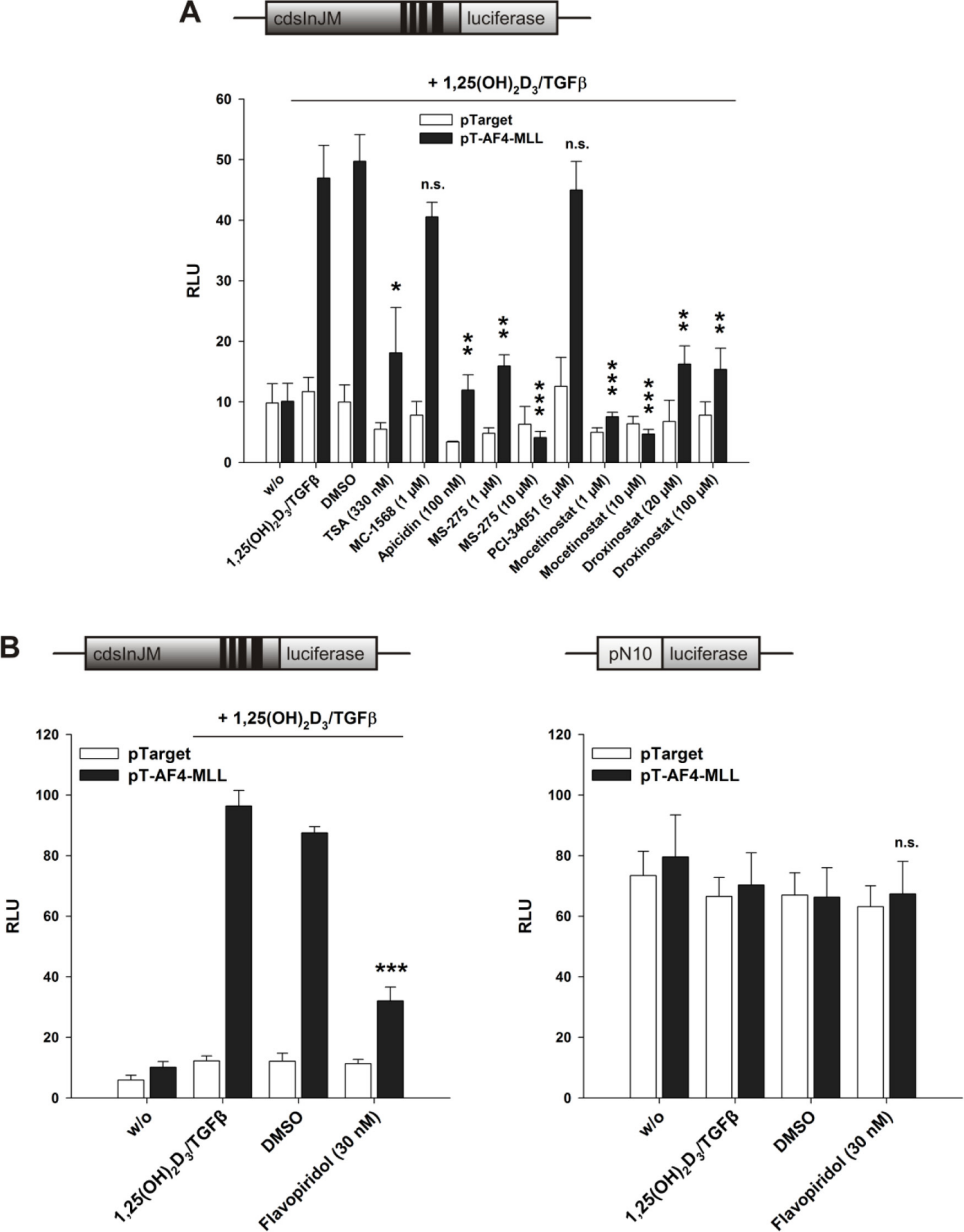
Effect of A. HDAC inhibitors and B. CDK9 inhibitor Flavopiridol on the AF4-MLL-dependent induction of reporter gene activity by calcitriol/TGFβ HeLa cells were transfected with the pGL3cdsInJM reporter gene construct plus the expression vectors for VDR and RXR (left panel) or the pN10 reporter gene construct (right panel). Additionally, cells were co-transfected either with the pTarget control vector or the expression vector for AF4-MLL as indicated. 16 hours after transfection, cells were incubated without or with calcitriol (50 nM) and TGFβ (1 ng/ml) and the indicated HDAC inhibitors or the CDK9 inhibitor Flavopiridol. After 40 hours, luciferase activity was determined. Results are given as RLU which include normalization to transfection efficiency. Each experiment was performed in triplicate. Results are presented as mean + S.E.M. of three independent experiments. Two-tailed *t*-test was performed to determine the significance of inhibitor treatment, indicated by asterisks (*P < 0.05, **P < 0.01, ***P < 0.001).

We have shown recently that Trichostatin A as well as HDAC class I inhibitors activate the human *ALOX5* promoter, but that Trichostatin A inhibits the calcitriol/TGFβ mediated 5-LO mRNA induction [4]. Since the calcitriol/TGFβ-mediated 5-LO mRNA induction is mainly associated with enhanced transcriptional elongation, we investigated here whether HDAC inhibitors modulate AF4-MLL-dependent stimulation of transcript elongation by calcitriol. To distinguish between the ‘transcript initiation’ and ‘transcript elongation’ processes, we used the CDK9 inhibitor Flavopiridol [24]. HeLa cells were transiently transfected with pGL3cdsInJM plus the VDR/RXR expression plasmids (Figure 8A and 8B, left graph) or the pN10 reporter plasmid (Figure 8B, right graph). Additionally, we included either pTarget (empty vector) or the expression vector for AF4-MLL as indicated. The cells were then incubated for 24 h with calcitriol/TGFβ, different HDAC inhibitors or Flavopiridol. The applied HDAC inhibitors show different selectivities for the various HDAC isoforms: MC-1568 is an inhibitor of class IIa HDACs; Apicidin inhibits HDAC1, HDAC2, HDAC3 and HDAC8; MS-275 (Entinostat) preferentially inhibits HDAC1, but also inhibits HDAC2 and HDAC3 at micromolar concentrations; PCI-34051 is a known HDAC8 inhibitor; Mocetinostat (MGCD0103) is an HDAC1, HDAC2 and HDAC3 inhibitor; Droxinostat is an HDAC3, HDAC6 and HDAC8 inhibitor [25–30]. Interestingly, the results revealed that class I HDAC inhibitors as well as Flavopiridol diminish the AF4-MLL-mediated induction of pGL3cdsInJM activity, which was associated with reduced 5-LO transcript elongation (Figure 8A and 8B, left graph). The strong inhibition of calcitriol/TGFβ-mediated induction of pGL3cdsInJM reporter activity by Flavopiridol supports earlier observations where the main effect exerted by the AF4-MLL fusion protein is the activation of transcriptional elongation [14]. Interestingly, Flavopiridol was not able to inhibit reporter gene activity with the *ALOX5* promoter plasmid pN10, suggesting that transcription from this particular plasmid is not P-TEFb-dependent (Figure 8B, right graph). This is supported by experiments where pN10 was co-transfected with either AF4- or AF4-MLL. Again, there was no stimulation of reporter gene activity by these plasmids, indicating that the process of ‘transcript initiation’ at the *ALOX5* promoter is not P-TEFb dependent, while the elongation control system residing within the *ALOX5* intron J-M region depends on P-TEFb. Furthermore, the reporter gene data suggest that HDAC class I inhibitors strongly block AF4-MLL-mediated 5-LO transcript elongation by calcitriol and TGFβ.

### Class I HDAC inhibitors and the CDK9 inhibitor Flavopiridol decrease 5-LO mRNA expression, protein expression and activity in MM6 cells

**Figure 9:**
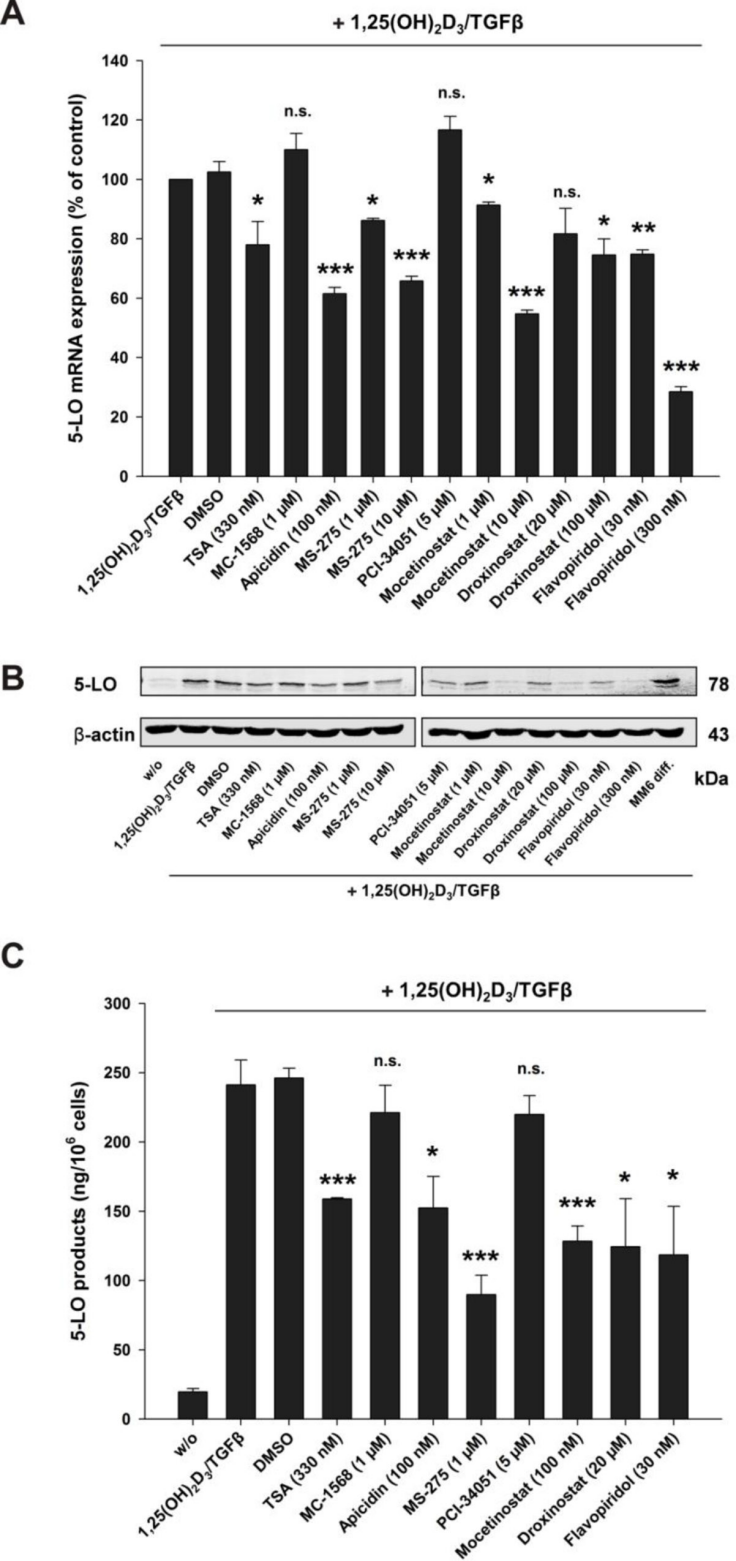
Inhibition of A. 5-LO mRNA expression, B. protein expression and C. activity by class I HDAC inhibitors or the CDK9 inhibitor Flavopiridol in MM6 cells MM6 cells were incubated without or with calcitriol (50 nM) and TGFβ (1 ng/ml) and in the presence of HDAC inhibitors or the CDK9 inhibitor Flavopiridol as indicated. After 24 hours, cells were harvested and 5-LO mRNA and protein expression were determined by real-time PCR and by Western blot analysis, respectively. The MM6 diff sample in panel B is derived from cells incubated for 96 hours with calcitriol/TGFβ. Cellular 5-LO activity was determined after cell differentiation by calcitriol/TGFβ in the presence of the indicated HDAC inhibitors for 96 hours. In each case three independent experiments were performed. Two-tailed *t*-test was performed to determine the significance of inhibitor treatment, indicated by asterisks (*P < 0.05, **P < 0.01, ***P < 0.001).

Next, we investigated the influence of HDAC inhibitors or Flavopiridol on 5-LO mRNA transcription, protein expression and enzyme activity in the human monocytic cell line MM6 cells. Previous data have shown that HDAC class I inhibitors stimulate the 5-LO promoter [4, 5] and here we found that these inhibitors block 5-LO transcript elongation by calcitriol/TGFβ. This is in line with the observation that the pan HDAC inhibitor TSA enhances basal but inhibits calcitriol/TGFβ-induced 5-LO expression in MM6 cells [4]. In order to investigate whether HDAC class I inhibitors are able to block 5-LO mRNA induction by calcitriol/TGFβ, MM6 cells were cultured without or with calcitriol/TGFβ in conjunction with the indicated inhibitors. As shown in Figure 9A, treatment of MM6 cells with HDAC1-3 inhibitors or with Flavopiridol caused a significant reduction of 5-LO mRNA expression in calcitriol/TGFβ-treated cells. As expected, inhibition of 5-LO mRNA expression by class I HDAC inhibitors and Flavopiridol also led to a decrease in 5-LO protein expression (Figure 9B) and to a remarkable inhibition of 5-LO enzyme activity (Figure 9C). This proved again, that the *ALOX5* gene that encodes for 5-LO, a key player for the inflammatory response in humans, is tightly regulated at the level of transcript initiation and elongation and that the inhibition of 5-LO mRNA expression in calcitriol/TGFβ-differentiated MM6 cells by HDAC class I inhibitors seems to be due to inhibition of transcript elongation.

### Class I HDAC inhibitors and the CDK9 inhibitor Flavopiridol decrease 5-LO mRNA expression in MV4-11 cells

**Figure 10:**
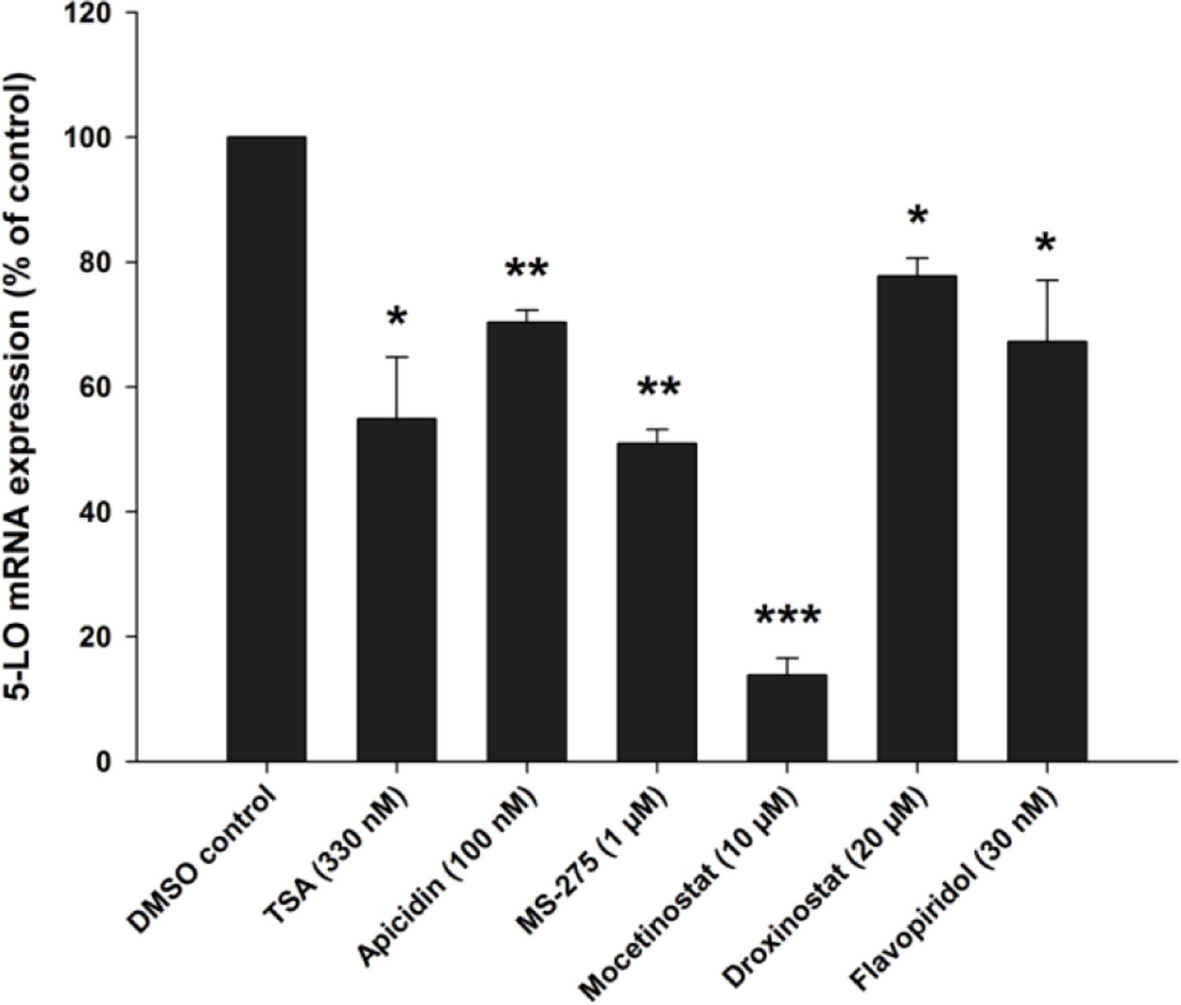
Inhibition of 5-LO mRNA expression by class I HDAC inhibitors or the CDK9 inhibitor Flavopiridol in MV4-11 cells MV4-11 cells were differentiated with (50 nM) and TGFβ (1 ng/ml) and cultured in the presence of HDAC inhibitors or the CDK9 inhibitor Flavopiridol as indicated. After 24 hours, cells were harvested and 5-LO mRNA expression was determined by real-time PCR in three independent experiments. One sample *t*-test was performed to determine the significance of inhibitor treatment, indicated by asterisks (*P < 0.05, **P < 0.01, ***P < 0.001).

Finally, we wanted to investigate the influence of HDAC inhibitors or Flavopiridol on a cell line carrying a t(4;11) chromosomal translocation. Therefore, the acute monocytic leukemia cell line MV4-11 was differentiated with calcitriol/TGFβ in the presence of the indicated HDAC inhibitors or Flavopiridol. Then, expression of 5-LO mRNA was analysed by real-time PCR. As shown in Figure 10, treatment of MV4-11 cells with HDAC1-3 inhibitors or with Flavopiridol caused a significant reduction of 5-LO mRNA expression and confirmed the data obtained with MM6 cells.

## DISCUSSION

5-LO is expressed in mature leukocytes which is in agreement with the function of LTs as mediators of innate immune reactions. During myeloid cell maturation, expression of 5-LO mRNA and protein is strongly upregulated by calcitriol and TGFβ [7, 20–22]. Initial studies demonstrated that this upregulation is not caused by 5-LO promoter activation [31] but by the stimulation of transcriptional elongation [8, 22].

However, at present, it is unclear how nuclear receptor signalling is linked with transcript elongation. AF4 was previously shown to be a key component of the super elongation complex which stimulates transcript elongation by recruiting P-TEFb (a heterodimer of CDK9 and cyclin T1) to arrested RNA polymerase II (RNAPII) and converts it into the elongating form by phosphorylation at Ser-2 of the carboxyterminal domain.

Here, we provide evidence that AF4 links nuclear receptor signaling with the process of transcriptional elongation. We demonstrate that the VDR/RXR heterodimer can directly interact with AF4 and that especially AF4-MLL supports transcript elongation in a vitamin D3 dependent manner. Of note, when we compared the effects of AF4 with AF4-MLL, the latter led to much higher vitamin D effects. It is described that the AF4-MLL protein accumulates in the cell, since it is resistant to proteasomal degradation [23]. Unfortunately, due to its short half-life, AF4 expression in the cells is low even after ectopic expression and it cannot be detected by Western blot. Consequently, we stabilized AF4 with the proteasome inhibitor MG132 and we observed that in the presence of MG132, AF4 exhibits similar induction of transcript elongation as AF4-MLL suggesting that the lower activity of AF4 mainly reflects its lower biological stability.

Calcitriol exclusively stimulates 5-LO transcript elongation but not initiation [8, 22]. This conclusion was further confirmed here with the P-TEFb inhibitor Flavopiridol which did not inhibit reporter gene activity when using the 5-LO promoter plasmid but strongly reduced the calcitriol/TGFβ-dependent induction of reporter activities with both elongation controlled reporter gene plasmids. From these experiments it became clear that all effects deriving from calcitriol/TGFβ signaling pathways directly affect the process of transcriptional elongation, but not the process of transcript initiation. The strong enhancement of the calcitriol effect in the elongation controlled reporter plasmids by coexpression of AF4 (after proteasome inhibition) or AF4-MLL suggests that the N-terminal part can mediate the stimulation of 5-LO transcript elongation by calcitriol. Of note, our data also suggest that the observed calcitriol effect depends on the presence of both nuclear receptors VDR and RXR, since only marginal effects of the ligand were observed when VDR/RXR was not co-expressed in our test system. This is supported by our coimmunoprecipitation experiments where a strong signal for AF4 was observed when the cells were co-transfected with both VDR and RXR. The strongest AF4 signal was obtained after immunoprecipitation by the VDR antibody and less by RXR antibody suggesting that the VDR might be the component of the VDR/RXR heterodimer which is responsible for the interaction with the AF4 complex. On the other hand one has to keep in mind that the different signal intensities could be simply due to different immunoprecipitation efficiencies of the VDR and RXR antibodies.

Taken together, we could demonstrate for the first time that vitamin D signaling is directly linked to transcript elongation. Indeed, careful review of the literature reveals that only a few of the vitamin D regulated genes show strong changes in transcription initiation (determined by nuclear run-off). In many cases there are only weak effects of calcitriol on transcription initiation that correspond to rather strong changes in steady state mRNA levels suggesting that upregulation of mRNA expression by calcitriol does not generally occur at the level of transcription initiation [32, 33]. As an example, changes in the steady state level of alkaline phosphatase mRNA was investigated in detail and attributed to the stabilization of newly synthesized transcripts [34].

Finally we analysed the influence of different HDAC inhibitors on 5-LO transcript elongation, as it is described that HDACs modulate the function of P-TEFb by deacetylation of certain lysine residues [35, 36]. Here we found that class I HDAC inhibitors abrogate the AF4-dependent activation of 5-LO transcript elongation by calcitriol/TGFβ in the respective reporter gene assays, as well as 5-LO mRNA and protein expression and cellular enzyme activity in Mono Mac 6 cells. These data suggest that deacetylation steps catalysed by class I HDACs are required for stimulation of transcript elongation. It was reported that the VDR/RXR heterodimer can recruit HDAC3 upon activation with calcitriol [37]. Thus, a possible explanation could be that recruitment of HDAC3 by VDR/RXR after activation by calcitriol mediates the stimulation of transcript elongation. However, additional studies will be necessary to clarify the exact mechanisms involved in the VDR/RXR-mediated regulation of transcript elongation.

Regarding leukemia development, our data confirm that the oncogenic translocation product AF4-MLL strongly supports enhanced transcript elongation. Interestingly, it has been recently shown that the AF4-MLL fusion protein is sufficient to induce ALL in mice and does not require the reciprocal translocation product MLL-AF4 [17]. Another study found that MLL-AF4 enhances the transcription of *RUNX1* which then cooperates with AF4-MLL. Thus, it could well be that elevated levels of RUNX1 protein render the MLL-AF4 fusion protein dispensable for the malignant transformation process. Under conditions of low RUNX1 expression, MLL-AF4 will be required to trigger the RUNX1/AF4-MLL pathway [19].

One hallmark of AF4-MLL is the functional hyperactivation of P-TEFb, which subsequently leads to enhanced transcript elongation by phosphorylation of RNA polymerase II at Ser2 of the CTD [14]. Our observation that HDAC class I inhibitors block aberrant transcript elongation caused by AF4-MLL suggests that HDAC class I inhibitors might be of considerable therapeutic interest for t(4,11)-leukemias. This is supported by our recent finding that class I HDAC inhibitors also attenuate the aberrant transcriptional activity of MLL-AF4 and strongly inhibit ALL cell growth [5]. Interestingly, these results are line with a recent investigation where gene expression profiling data of t(4;11) leukemia cells were related to connectivity maps. This type of analyses revealed that MLL leukemia cells could profit from HDACi [38]. Here, we provide the molecular mechanism how HDAC inhibitors counteract the oncogenic functions of the AF4-MLL fusion proteins on transcript elongation. Taken together, the present data suggest that HDAC class I inhibitors might have a considerable potential for the treatment of t(4, 11) leukemias.

## MATERIALS AND METHODS

### Cells and cell culture

HeLa cells (DSMZ no. ACC-57) and HEK293T cells (DSMZ no. ACC-635) were cultured at 37°C in a humidified atmosphere with 5% CO_2_ in Dulbecco's modified Eagle medium (DMEM) supplemented with 10% (v/v) fetal calf serum (FCS), L-glutamine (2 mM), sodium pyruvate (1 mM), penicillin (100 U/ml) and streptomycin (100 μg/ml).

Mono Mac 6 (MM6) cells (DSMZ no. ACC-124) were grown in RPMI 1640 medium supplemented with 10% (v/v) FCS, L-glutamine (2 mM), 1× non-essential amino acids, sodium pyruvate (1 mM), oxalacetate (1 mM), penicillin (100 U/ml), streptomycin (100 μg/ml) and insulin (10 μg/ml). MV4-11 cells (DSMZ no. ACC-102) were grown in RPMI 1640 medium supplemented with 10% (v/v) heat inactivated FCS. Differentiation of MM6 and MV4-11 cells was carried out at 37°C in a humidified atmosphere of 6% CO_2_.

### Plasmids

The 5-LO promoter firefly luciferase reporter gene vector pN10, containing the ALOX5 promoter sequence from −778 to +53 (relative to the transcriptional start site), was described previously [4, 31]. The reporter gene constructs pN10cdsInJM and pGL3cdsInJM, containing coding sequence (cds) and the last four introns of the *ALOX5* gene, were obtained by PCR amplification using genomic DNA from MM6 cells, which was already described elsewhere [39]. Expression plasmids for the human vitamin D receptor (pSG5-VDR) and retinoid X receptor alpha (pSG5-RXR) were kindly provided from C. Carlberg (Kuopio, Finland). The renilla luciferase control vector pRL-SV40 was purchased from Promega. Expression vectors for full-length AF4, MLL, AF4-MLL and MLL-AF4 were gained as recently described [5]. All expression cassettes are flanked by the rare-cutting Sfi1 sites and were cloned in a Sfi1 restriction site-modified pTarget vector (Promega, Mannheim, Germany) [40].

### Reagents

Calcitriol (17936) and the proteasome inhibitor MG132 (Z-Leu-Leu-Leu-al, C2211) were purchased from Sigma-Aldrich, Schnelldorf, Germany. Human TGFβ1 was purified from platelets according to [41]. TSA (Sigma-Aldrich: T1952), MC-1568 (Sigma-Aldrich: M1824), Apicidin (Sigma-Aldrich: A8851), MS-275 (Enzo Life Sciences, Lörrach, Germany: ALX-270-378-M005), PCI-34051 (Cayman Chemical, Ann Arbor, MI, USA: 10444), Mocetinostat (Selleckchem, München, Germany: S1122), Droxinostat (Selleckchem: S1422) and Flavopiridol (Sigma-Aldrich: F3055) were all dissolved in DMSO (AppliChem, Darmstadt, Germany: A3006). AFF1 ELISA Kit (E98872Hu) was obtained from USCN Life Science, Wuhan, Hubei, PRC.

### Calcium phosphate transfection and reporter gene assay

24 hours prior to transfection, HeLa cells were seeded at a density of 4 × 10^4^ cells per well in 24-well plates by using 1 ml of the cell line specific medium without phenol red. Transient transfection of the cells with 225 ng 5-LO firefly luciferase reporter gene construct (pN10, pN10cdsInJM or pGL3cdsInJM), 225 ng expression vector (pTarget, pT-AF4, pT-MLL, pT-AF4-MLL or pT-MLL-AF4), 50 ng nuclear receptor expression vector (pSG5 or pSG5-VDR/pSG5-RXR, pCGN or pCGN-SMAD3/pCGN-SMAD4) and 20 ng renilla luciferase control vector (pRL-SV40) per well was performed by calcium phosphate precipitation method [42]. The medium was changed 16 hours after transfection and the cells were incubated without or with 50 nM calcitriol and 1 ng/ml TGFβ and additionally with inhibitors, if indicated in the figures. 40 hours after transfection firefly and renilla luciferase activity was determined with the Dual-Glo^®^ Luciferase Assay System (Promega) and a TECAN infinite^®^ M200 luminometer. Data were calculated as relative light units (RLU) by normalization of transfection efficiency with the values of renilla luciferase activity in at least three independent experiments.

### Stable knockdown of AF4 in HeLa cells by lentiviral transduction

MISSION shRNA plasmid for AF4 knockdown (NM_005935.1-3282s1c1: TRCN0000021975) was obtained from Sigma-Aldrich. HEK293T cells were transfected with 10 μg shRNA plasmid, 6.5 μg lentiviral packaging vector pCMV-dR8.91 and 3.5 μg envelope plasmid pMD2.G by calcium phosphate precipitation method. The medium was changed 4 hours after transfection and supernatant was collected after 72 hours. For transduction, 4 × 10^4^ HeLa cells in 1 ml medium per well were prepared in 24-well plates and were treated after 24 hours with 4 μg/ml protamine sulfate and 10, 50, 100 or 500 μl supernatant including lentiviral particles. The cells were centrifuged (90 min, 2500 rpm, 32°C) and cultured for 72 hours. Selection of transduced cells was performed by puromycin (0.75 μg/ml) and knockdown efficiency was checked by quantitative PCR analysis with a StepOnePlus™ System (Life Technologies, Darmstadt, Germany). The sequences of the AF4 primers were 5′-ATGTCCGGCCCATGGAT (forward) and 5′-GGCAGGCACTTTCAAGTCTGT (reverse). Results were normalized to β-actin Ct values. Sequences of the β-actin primers were as follows: 5′-CGGGACCTGACTGACTACCTC (forward) and 5′-CTTCTCCTTAATGTCACGCACG (reverse). Each sample was set up in triplicates. The mRNA expression was quantified by comparative ΔΔCT method with three independent real-time PCR experiments. Knockdown of AF4 protein expression was quantified in three independent experiments via AFF1 ELISA Kit according to the manufacturer's protocol.

### Co-IP experiments

1 × 10^7^ HEK293T cells were transiently transfected with 10 μg of the expression plasmid for AF4 and 5 μg each of the expression plasmids for the nuclear receptors VDR and/or RXR and cells were grown for 48 hours. For immunoprecipitation, nuclear extracts were normalized to 1 mg aliquots of total protein concentration per antibody. All samples were pre-cleared by addition of 1 μg non-specific IgG and 20 μl Protein-G-Agarose (Santa Cruz Biotechnology) and incubated for 30 min at 4°C under rotation. Supernatants were collected and 1 μg of RXR antibody (Santa Cruz: sc-553) and/or VDR antibody (Santa Cruz: sc-1008) were added and further incubated for 1 hour. 25 μl of Protein-G-Agarose were added and incubated for additional 2 hours. Pellets were washed three times with lysis buffer and eluted by boiling for 5 minutes in 2 x Laemmli-buffer. Input and eluate samples were separated by SDS-PAGE and analysed by Western blot analysis for RXR, VDR and AF4.

### Real-time PCR analysis

MM6 cells or MV4-11 were seeded at a density of 2 × 10^5^ cells/ml and incubated for 24 hours without or with 50 nM calcitriol, 1 ng/ml TGFβ and with the indicated inhibitors. For real-time PCR total RNA was extracted from the cells by the Total RNA Kit from Omega Bio-Tek (Norcross, GA, USA: R6834-02) including DNase I digestion. 2 μg RNA was reverse transcribed into cDNA using High-Capacity cDNA Reverse Transcription Kit (Life Technologies: 4368814) according to the manufacturer's protocol. Quantitative PCR analysis was performed with a StepOnePlus™ System (Life Technologies). The sequences of the 5-LO primers were 5′-GTTCCTGAATGGCTGCAAC (forward) and 5′-GGCAATGGGGACAATCTTG (reverse). Results were normalized to β-actin Ct values. Sequences of the β-actin primers were as follows: 5′-CGGGACCTGACTGACTACCTC (forward) and 5′-CTTCTCCTTAATGTCACGCACG (reverse). Each sample was set up in triplicates. The expression was quantified by comparative ΔΔCT method of three independent experiments.

### Western blot analysis

MM6 cells were seeded at a density of 2 × 10^5^ cells/ml and incubated for 24 hours without or with 50 nM calcitriol, 1 ng/ml TGFβ and with the indicated inhibitors. Whole cell lysates were then separated by SDS-PAGE and were transferred to nitrocellulose membranes (Amersham Hybond™-C Extra, GE Healthcare, Freiburg, Germany). After 1 hour blocking, the membranes were incubated with the appropriate primary antibody against 5-LO (BD Biosciences, Heidelberg, Germany: 610695) and β-actin (Santa Cruz: sc-1616) overnight at 4°C. The membranes were washed with PBS/0.1%-Tween-20 and were incubated with infrared-dye conjugated secondary antibodies (LI-COR Biosciences, Bad Homburg, Germany) for 45 minutes at room temperature. Detection of the protein signals was performed with the Odyssey^®^ Infrared Imaging System (LI-COR Biosciences).

### 5-LO activity assay

MM6 cells were seeded at a density of 2 × 10^5^ cells/ml and were incubated without or with 50 nM calcitriol, 1 ng/ml TGFβ and in the absence or presence of HDAC inhibitors for 96 hours. For determination of 5-LO product formation of intact cells, 3 × 10^6^ cells were resuspended in 1 ml PGC buffer (PBS containing 1 g/l glucose and 1 mM CaCl_2_). The reaction was started by the addition of calcium ionophore A23187 (2.5 μM) and exogenous arachidonic acid (20 μM). After 10 minutes at 37°C the reaction was stopped by addition of 1 ml methanol, 30 μl of 1N HCl, 500 μl of PBS and 200 ng of prostaglandin B1 (internal standard). Finally, 5-LO metabolites were extracted by a C-18 solid-phase extraction column and analyzed by HPLC as described previously [43]. 5-LO product formation was determined as the amount of 5-LO products measured as nanograms produced per 10^6^ cells, which include leukotriene B4 (LTB4), the all-trans-isomers of LTB4 and 5-hydroxyeicosatetraenoic acid (5-HETE). Each experiment was performed at least three times for calculation of mean and SEM.

### Statistics

Results were calculated as mean ± S.E.M. of at least three independent experiments. Two-tailed Student's *t*-test was performed to determine the significance between two groups. One sample *t*-test was performed to analyse significance of AF4 knockdown. Thereby, statistical significance was illustrated as follows: *P < 0.05, **P < 0.01, ***P < 0.001.
